# sTetro-Deep Learning Powered Staircase Cleaning and Maintenance Reconfigurable Robot

**DOI:** 10.3390/s21186279

**Published:** 2021-09-18

**Authors:** Balakrishnan Ramalingam, Rajesh Elara Mohan, Selvasundari Balakrishnan, Karthikeyan Elangovan, Braulio Félix Gómez, Thejus Pathmakumar, Manojkumar Devarassu, Madan Mohan Rayaguru, Chanthini Baskar

**Affiliations:** 1 Engineering Product Development Pillar, Singapore University of Technology and Design (SUTD), Singapore 487372, Singapore; rajeshelara@sutd.edu.sg (R.E.M.); selvasundarisubramanian@gmail.com (S.B.); e_karthikeyan@sutd.edu.sg (K.E.); brauliofelixgomez@gmail.com (B.F.G.); pathmakumar_thejus@mymail.sutd.edu.sg (T.P.); manojkumar@sutd.edu.sg (M.D.); 2 Department of Electrical Engineering, Delhi Technological University, Delhi 110042, India; mmrayguru87@gmail.com; 3 School of Electronics, Vellore Institute of Technology Chennai, Vellore 600127, India; chanthini.baskar@vit.ac.in

**Keywords:** staircase maintenance, reconfigurable robot, sTetro, deep learning, SSD MobileNet, cleaning robot

## Abstract

Staircase cleaning is a crucial and time-consuming task for maintenance of multistory apartments and commercial buildings. There are many commercially available autonomous cleaning robots in the market for building maintenance, but few of them are designed for staircase cleaning. A key challenge for automating staircase cleaning robots involves the design of Environmental Perception Systems (EPS), which assist the robot in determining and navigating staircases. This system also recognizes obstacles and debris for safe navigation and efficient cleaning while climbing the staircase. This work proposes an operational framework leveraging the vision based EPS for the modular re-configurable maintenance robot, called sTetro. The proposed system uses an SSD MobileNet real-time object detection model to recognize staircases, obstacles and debris. Furthermore, the model filters out false detection of staircases by fusion of depth information through the use of a MobileNet and SVM. The system uses a contour detection algorithm to localize the first step of the staircase and depth clustering scheme for obstacle and debris localization. The framework has been deployed on the sTetro robot using the Jetson Nano hardware from NVIDIA and tested with multistory staircases. The experimental results show that the entire framework takes an average of 310 ms to run and achieves an accuracy of 94.32% for staircase recognition tasks and 93.81% accuracy for obstacle and debris detection tasks during real operation of the robot.

## 1. Introduction

With the advent of the 21st century, autonomous robots have become an integral part of intelligent machines for performing various tasks including inspection, manufacturing, warehouse handling, and maintenance (cleaning, dusting and painting etc.). Among them, autonomous cleaning robots play a vital role in many day to day activities and also in industrial automation applications [1,2]. These robots have become the need of the day due to the large demand in household works, hospitals and industries. There are many commercially available autonomous cleaning robots in the market. However, these robots are designed for single floor operations and their mechanism cannot support staircase navigation to reach the next floor. They are also not capable of cleaning the steps of the staircases [3]. Various staircase climbing robotic architectures have been proposed for different applications. These include staircase climbing wheelchairs [4,5,6], rescue operations in disasters [7,8] and security applications [9]. While there are many such applications, very few works have been reported on the design of cleaning robots with staircase climbing capabilities. Takahisa et al. [10,11] proposed an autonomous cleaning robot suitable for both flat surfaces and staircases. in this work, an L-shaped leg was designed to climb down the staircase. A vertical climbing mechanism based staircase cleaning robot is reported by Rajesh et al. [12]. In [3,13], the authors propose a modular re-configurable robot sTetro for staircase cleaning where the robot is equipped with a vertical conveyor mechanism for climbing the staircase. Generally, these robots use an array of sensors such as 2D LiDAR and 1D laser sensors [3,13] and Position Sensitive Detectors (PSDs) [10,11] to recognize their environment. However, these sensors only provide limited information about the environment due to their limited capability when it comes to recognizing the object type. In addition, they are not capable of detecting obstacles or debris, which is crucial for safe operation and efficient cleaning.

Computer vision based environmental perception has been widely deployed for various autonomous robotic platforms [14,15,16,17]. These methods are cost effective and are capable of operating in a wide range of scenarios. Instead of traditional computer vision based techniques, learning based environmental perception (e.g., Deep Learning) is a new paradigm. The deep learning neural network layers can learn features to distinguish and determine objects at high accuracy and can be used in various vision tasks such as outdoor environmental perception for autonomous vehicles [18,19,20], cleaning and maintenance robot vision pipeline [2,21,22,23,24], mobile robot place recognition and mapping tasks [25,26,27,28]. While these state-of-the-art deep learning algorithms have achieved impressive results for environment perception design, there are some shortcomings such as false detection, which can be due to objects with similar features. This can lead to inconsistencies during real-time robotic applications. One approach to solve this issue is by using additional data, such as depth information.

Recently, many works have been reported with depth based fusion to overcome the false detection of objects and improve the model’s classification accuracy [29,30,31,32,33]. In [31,32] authors propose a RGB-D based object detection technique and describe how to utilize the depth information to effectively enhance the detection quality. In these implementations, the first one adds the fourth channel for depth directly, and then equally convolutes all channels in one network [31]. The other separately processes the depth and color (RGB) using two independent networks [32]. Tanguy et al. [33] fused the depth information and RGB data to increase the detection performance of the YOLOv2 object detection framework. Here, the author introduced a fusion layer architecture for combining the depth and RGB detection networks. Even though many works have explored the advantages of using deep learning techniques for detection applications, none of these works are targeted towards environmental perception for staircase cleaning robots.

This work proposes an operation framework empowered by the deep learning based environmental perception system for our modular re-configurable staircase cleaning robot sTetro [3,13]. The environmental perception system assists the robot in makings decisions, such as recognizing the staircases for autonomous stair climbing operation, static and dynamic obstacle detection for safe navigation and debris detection for efficient cleaning.

The flow of the paper is organized as follows: The architecture of the sTetro platform is detailed in Section 2 and Section 3, which describe the proposed EPS and autonomous staircase climbing scheme. This is followed by the experimental setup and results in Section 4. Finally, Section 5 describes the conclusions and future work.

## 2. Brief Overview of sTetro

**Figure 1:**
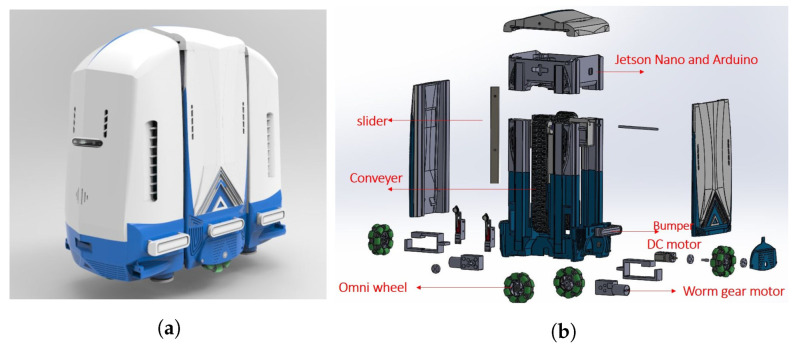
(**a**) sTetro Overview & (**b**) Exploded view.

**Figure 2:**
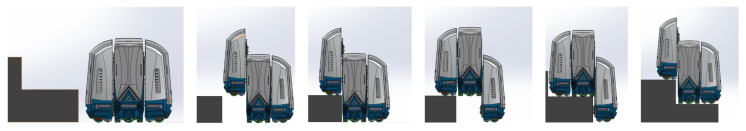
sTetro climbing mechanism.

sTetro is a modular re-configurable cleaning and maintenance robot developed by our ROAR lab. The general view and exploded view of sTetro are shown in Figure 1a,b. Its body is comprised of three cuboids. The slider mechanism mounted on both the front and the back of the central (second) cuboid interconnects with the first and third cuboids. Each cuboid is a vertical block with hollow space which contains the control circuits, debris collection chamber, suction fan, and sweeping mechanism. The vertical conveyor belt mechanism enables the robot to climb the staircase as shown in Figure 2. The limitation of being able to clean only a single floor is overcome by modular design with a re-configurable mechanism.

### Hardware and Software Description

**Figure 3:**
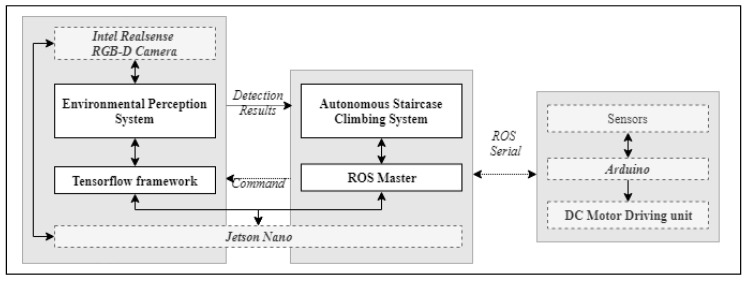
Hardware architecture.

sTetro hardware architecture is comprised of Jetson Nano hardware module with RGB-D camera, Arduino with DC motor control unit and sensor unit as shown in Figure 3.

Here, the Jetson Nano module acts as a Central Control Unit (CCU) which controls the entire operation of the robot. The module is comprised of ARM A57 CPU and 128 Core maxwell GPU with 4GB memory for real-time deep learning inference and running on Ubuntu 18. The processing unit utilizes Rosserial communication interface to enable the communication between ROS nodes, and other modules include environmental perception unit and Arduino control blocks. This bridge is used to communicate the sensor data and trajectory information between Arduino Mega microcontroller and Jetson Nano unit.

Arduino mega microcontroller performs the locomotion control and onboard sensor interface. It collects the data from the various sensor modules and sends it CCU. The control signal to the DC motor unit based on the received trajectory information is generated using inbuilt PWM module.

**Figure 4:**
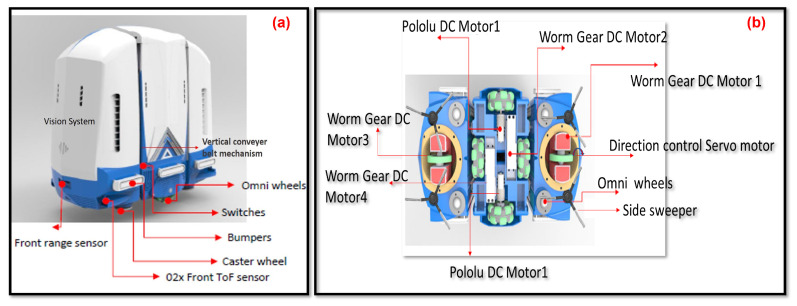
(**a**) Sensor assembly. (**b**) Locomotion DC motor assembly.

Various sensors are integrated to perform the self-reconfiguration and locomotion tasks (Figure 4a). Bump sensors were used to detect the inner string of the staircase, and six bump sensors were assembled on both the left and right sides of the robot. For staircase riser detection, we provide two Time Of Flight distance sensors (TOF) mounted on the front face of the first block, and two mechanical limit switches assembled in front of the second and third blocks. Furthermore, at the bottom of all three blocks, we provide Time Of Flight distance sensors (TOF) to detect the staircase runner on each step while climbing. The detail of motor and all sensor detail and its communication protocol is given in Table 1.

Figure 4b shows the locomotion unit assembly. Adafruit servo shields are used to drive the four high torque servo motors. Of these four servos, two of them lift the three blocks through the vertical conveyor belt mechanism. The remaining two servo motors are used to drive the direction control mono-wheels fitted in the first and third blocks. In addition, the driving unit also drives six DC motors: Four worm gear DC motors and two Pololu motors. These motors are used to drive the Omni-direction wheels, which were used to navigate the robot in planar directions. These motors are operated at 12 volts and consume an average of 800 mA current from the power source.

Four side sweepers are used to accomplish the cleaning task and are mounted on Modules 1 and 3 and propelled by 12v DC with a 100 RPM Metal gear motor. Side sweepers spin inwards to grab the dust particle and push towards the suction mouth; then the suction unit collects to the collection chamber.

## 3. Proposed Framework

**Figure 5:**
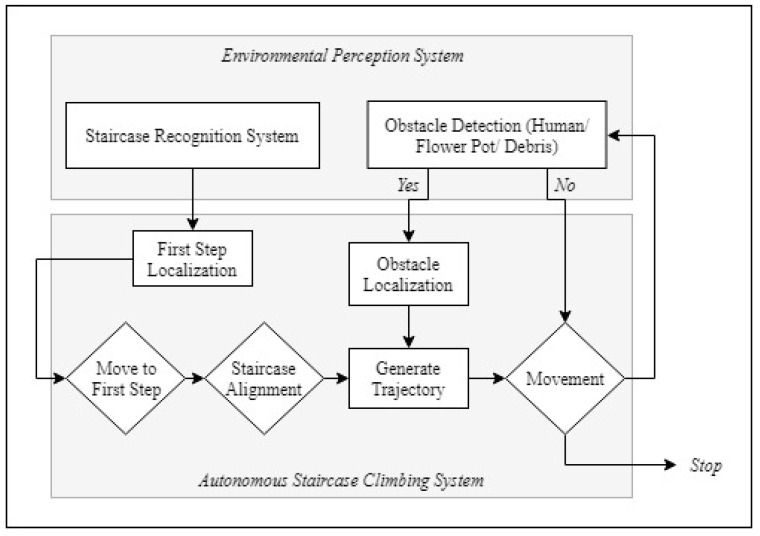
Proposed Scheme.

The previous work suggests the design principles behind the sTetro platform along with a basic sensor based climbing mechanism [3,13]. This work enhances the sTetro performance by incorporating the deep learning based environmental perception system, which is utilized to deploy the autonomous staircase climbing framework. Figure 5 Shows the functional block diagram of the proposed scheme. It comprises the environmental perception system and the autonomous stair climbing framework. A detailed description of the two is given below.

### 3.1. Environmental Perception System (EPS)

Environmental Perception System plays a crucial role to enable autonomous control systems in the robot. This system is tasked to recognize the objects in the environment. The EPS system is comprised of the RGB-D vision sensor, SSD MobileNet based object detection module and depth based error correction unit. The EPS addresses the key challenges of staircase cleaning robot including detection of staircases, debris (e.g., liquid spillages), static and dynamic obstacles present on the stairs (e.g., flower pots, human) which may obstruct the path of the robot during climbing. In the current study, the vision sensor data was used to identify the human and plant pot on the staircase through a deep learning algorithm. According to obstacle position the robot navigation direction will be controlled by CCU.

#### 3.1.1. Object Detection

**Figure 6:**
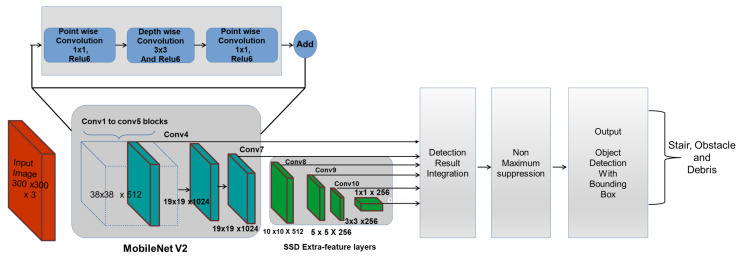
SSD MobileNet V2 Object Detection Framework.

The SSD-MobileNet v2 object detection framework [34,35] is used to enable real-time object detection. Here, Mobilenet V2 is the base network called the feature extractor and SSD is the object localizer. Figure 6 shows the schematic representation SSD-MobileNetv2 object detection framework. The MobileNet feature extractor extracts the high-level features from the captured image stream and generates a feature map, which describes the important features needed for classification or detection tasks. The detection model, SSD uses the feature map and detects the class of an object and its location using a bounding box. The framework is trained to identify staircases, debris and obstacles on the staircase such as humans and flower/plant pots.

Mobilenet v2 is a lightweight feature extractor, suitable for use in real-time object detection application and low power applications of embedded devices. Its architecture is comprised of multiple residual bottleneck layers. These layers use 3×3 depthwise convolution layers, which are more efficient compared to standard convolution layers. They also employ 1×1 convolutions instead. The architecture also uses ReLU6 layers, which are ReLU layers with an upper limit of 6. The upper limit prevents the outputs from scaling to a very large value, thereby also reducing the computation cost. Moreover, the model gives better accuracy for the architecture due to the residual connection over the non-residual architectures.

SSD is an object localizer which runs on top of the MobileNet v2 feature extractor. The network utilizes output from the last few layers (feature map) from the feature extractor to predict the location and class of objects. In the SSD architecture, the input is passed through different convolution layers of different sizes. These layers decrease in size progressively through the network. The purpose of these layers is to enable the network to detect objects of different shapes and sizes. A fixed set of predictions is taken from each one of these convolution layers and merged at the end. Furthermore, the computational cost of the model is reduced by choosing the fixed set of shapes. The model outputs locations and confidence of various objects in the image.

The loss for each prediction is computed as the combination of confidence loss $L_{confidence}$ and location loss $L_{location}$ (Equation (1)). The error in the prediction of class and confidence is termed as confidence loss and location loss is calculated as squared distance between the coordinates of prediction. The parameter α balances both the losses and reduced the influence on overall loss. Root Mean Squared gradient descent algorithm [36] is used to optimize the loss. This algorithm computes the weights $w_{t}$ at any time *t* using the gradient of loss *L*, $g_{t}$ and gradient of the gradient $v_{t}$ (Equations (2)–(4)). The Hyperparameters β,η are used to balance the momentum and gradient estimation and ϵ is a minimum value close to zero for preventing divide-by-zero errors.

(1) $$L=\frac{1}{N}\left(L_{confidence}+\alpha L_{location}\right)$$

(2) $$v_{t}=\beta v_{t-1}+\left(1-\beta \right)g_{t}^{2}$$

(3) $$\Delta w=\frac{-\eta }{\sqrt{v_{t}+\epsilon }}\times g_{t}$$

(4) $$w_{t+1}=w_{t}+\Delta w$$

#### 3.1.2. Depth Based False Detection Correction

**Figure 7:**
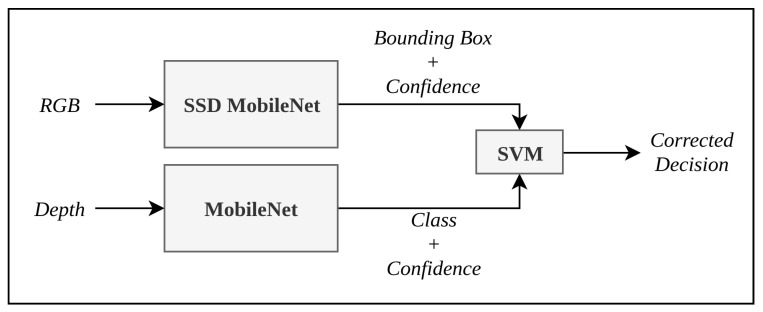
False detection correction using depth.

The detection of the staircase through the use of SSD MobileNet model using RGB data leads to false detection of objects with features similar to staircases. This may include objects similar to staircase such as patterned walls, railings, benches, etc. However, the depth data can be used in tandem to correct the classification decision of the SSD MobileNet. The depth information is fed through the MobileNet network architecture which classifies whether a given object is a staircase or not. However, the depth information may be noisy which may also lead to false detection. Hence, the two outcomes are fused using a support vector machine (SVM) to identify staircases with higher accuracy. Figure 7 shows the model used to identify staircases.

SVM determines a hyperplane which separates the different classes present in the dataset [37]. The position of a point relative to this hyperplane determines its class. In this work, a soft margin SVM is used, whose hyperplane boundary is computed by minimizing the function f(x) as computed in Equation (5).

(5) $$f\left(x\right)=\left[\frac{1}{n}\times \sum max\left(0,1-y_{i}\left(\vec{m}\cdot \vec{x_{i}}-b\right)\right)\right]+\lambda ||\vec{m}{\left|\right|}^{2}$$

where $\left|\right|\vec{m}{\left|\right|}^{2}$ is a hyper parameter which dictates the size of the soft margin. Due to the linear inseparability of the data, an RBF kernel is used to reduce the dataset into a linear space. The kernel uses the kernel function *K* on $x_{1}$ and $x_{2}$ using a free parameter σ as shown in Equation (6).

(6) $$K\left(x_{1},x_{2}\right)=\exp\left(-\frac{\left|\right|x_{1}-x_{2}{\left|\right|}^{2}}{2\sigma^{2}}\right)$$

### 3.2. Autonomous Staircase Climbing Methodology for sTetro

For autonomous staircase climbing, three crucial environmental features are considered. First, the system should detect staircases, which is used to plan the locomotion to reach the staircase. Next, the robot must detect any obstacles present on the steps which can prevent its operation in that specific region. This includes static and dynamic obstacle like flower/pot plants or humans (a possible obstacle in staircase), with which the robot should not collide. Finally, the robot must move slowly in the slippage region (here we consider liquid spillage debris) and also avoid the slippage region to perform the climbing operation.

#### 3.2.1. First Step Identification and Align with Staircase

A combination of the RGB and depth information is used to determine the location of the first step of the staircase and the angle at which the robot is facing the staircase. This information is crucial to guide the robot towards the staircase. A point closer to the center of the step is chosen to allow room for the robot to align itself and start climbing. Common techniques for detection of steps involve the use of edge detection techniques such as Canny [38] or Sobel [39]. This is followed by line detection using Hough Transform [40]. However, steps can be straight or curved, which limits the accuracy of the Hough transform technique. Furthermore, Hough Transform is highly influenced by noise. One option to alleviate the issue of curved steps is to use contour detection algorithms. These form arbitrary lines by joining points through a Square sweep, Moore sweep or Radial sweep. These check nearby squares and their neighbors in a particular manner. However, for the case of step detection, the change of gradient between one edge point and the next is very small. Hence, search for the next point can prioritize the direction of the gradient over other directions. Furthermore, staircases often have very small gradients, which can narrow the search region at each point.

This work proposes a contour detection algorithm specifically for the case of first step recognition (Algorithm 1). The algorithm gives a higher preference to points along the gradient. This prevents the contour detected from being highly influenced by noise. Moreover, the algorithm checks for points which have deviated from the gradient. Due to this, the contour detected by this technique can be curved or straight, thereby allowing efficient use in all types of staircases.
**Algorithm 1:** Contour detection algorithm
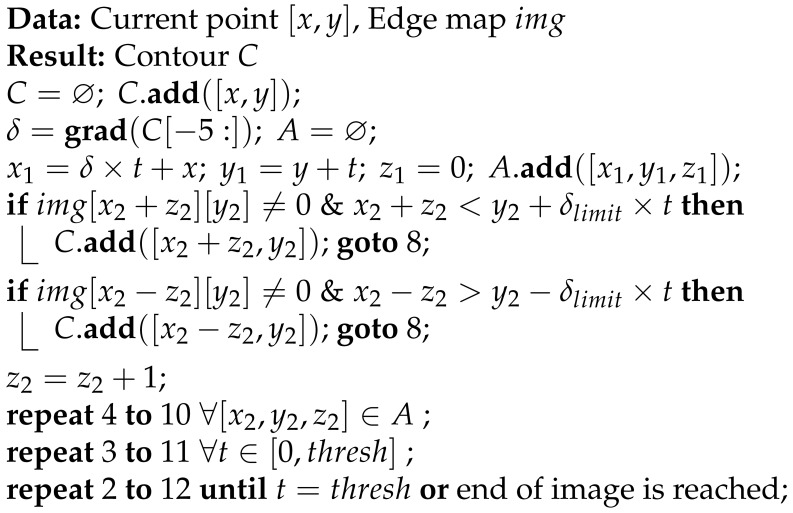


The algorithm generates the contour representing the first step from the edge map img. The algorithm is run at each edge point x,y from the bottom to top, left to right in the edge map. The function **add** added a point to the start of an array. In the algorithm, the gradient δ of the five most recent detected points in the contour is computed through function grad. This function computes the gradient by using regression to fit a line through the given points. If the gradient is too large, the function limits the gradient to $\delta_{limit}$, since steps tend to have small gradients. In each iteration, a new point along a gradient is explored, whose coordinates are $x_{1}$ and $y_{1}$. The previously checked points, which are present in the array *A*, are also checked but for larger deviation from the gradient as compared to previous iterations ($z_{2}$), until the deviation gradient limit $\delta_{limit}$ is reached. This is repeated until a search threshold thresh is reached. This defines the maximum distance that can exist between two edge points. The whole process is repeated until no new edge points are found (t>thresh) or the end of the image is reached. Finally, contours of width lesser than half of the image are ignored and the algorithm is re-run in this case.

After determining the contour, the midpoint of the first step is determined. First, a line is fitted through the points close to the horizontal midpoint of the bounding box by regression. The misalignment angle is determined through the slope of this line. Further, the intersection of this line and the horizontal midline is the midpoint of the first step. The distance to travel to reach the staircase is determined through the depth information of this location in the depth map.

#### 3.2.2. Obstacle and Debris Detection and Localization

Obstacle and debris detection task is accomplished through SSD MobileNet object detection framework. Obstacles can cause collisions or slippages when climbing the staircase. Similarly, the debris detection result assists the robot to perform efficient cleaning. Here, stains and liquid spillage are considered as debris which is commonly found debris in the staircase environment. Furthermore, it is crucial to determine the step in which the obstacle/debris is present in order to plan the appropriate cleaning trajectory. The depth information corresponding to the detected object is used to determine this. The depth data is grouped into clusters through the use of k-means clustering, which is used to determine which step an obstacle/debris is in. In most of the staircases, four steps are clearly visible from the robot perspective; that is why four centers are chosen for the algorithm.

#### 3.2.3. Trajectory Planning

**Figure 8:**
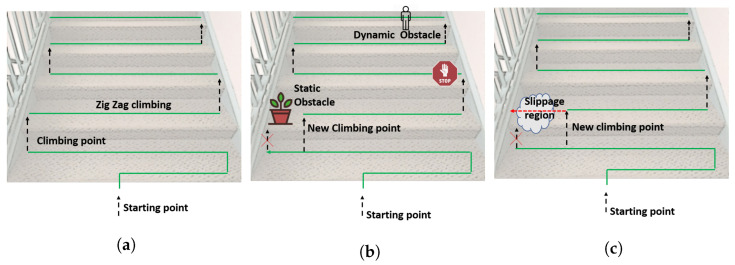
Trajectory planning, (**a**) zig-zag trajectory, (**b**) avoids obstacles while climbing, (**c**) avoid the slippage region.

While climbing, the robot generates a zig-zag trajectory (Figure 8a) completely covering the staircase. The algorithm avoids obstacles (Figure 8b) while climbing. Further, the robot must move slowly in the slippage region (Figure 8c) and also avoid the slippage region while performing the climbing operation. The robot performs the climbing operation next to recognizing the slippage area (Figure 8c) and also stops the cleaning task (Figure 8b) on human detection. Algorithm 2 defines the trajectory planning scheme. Function **detect** updates the staircase data *S* representing the position of obstacles and debris. Static obstacles (pots) such as debris are retained in the step data through iterations (represented by ′*O*′, ′*D*′) and dynamic obstacles (humans) are maintained temporarily (represented by ′*H*′). ${}^{\prime }E^{\prime }$ represents that the position is empty. Function climb performs the step climbing action, as shown in Figure 2 in Section 2. Function move (*M*) moves the robot in direction *M*. Sleep (*t*) pauses the robot operation for *t* seconds.
**Algorithm 2:** Trajectory planning
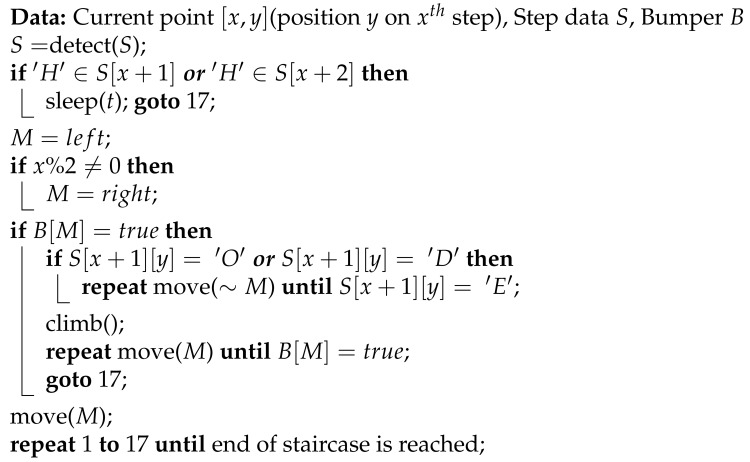


## 4. Results and Discussion

**Figure 9:**
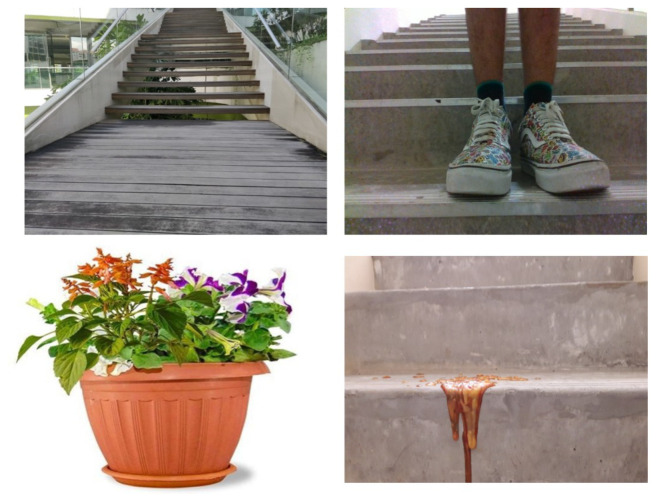
Dataset.

This section describes the experimental results of the proposed scheme. To initiate the experiment, the dataset was generated by capturing images of the staircase, obstacles, and debris (stain and liquid spillage) using Intel’s Real Sense RGB-D camera in robot perspective. Each class has about 2600 images. Figure 9 shows some of the images present in the dataset from each class.

### 4.1. Performance Metrics

Standard performance metrics (accuracy, precision, recall and $F_{1}$ scores) are calculated to estimate the performance of the model. K-Fold cross validation is used in this work. The dataset is divided into 10 parts and nine parts are used for training, while one part is used for testing. The same process is repeated 10 times to ensure all possible combinations.The above process removes any biasing conditions due to the training and testing dataset. Accuracy and precision are estimated for each task. The model was trained using the TensorFlow framework on Ubuntu 16.04 with the following hardware configuration: Intel Xeon E5-1600 V4 CPU, 64 GB RAM and an NVIDIA Quadro P4000 GPU with 12 GB video memory. Run time results are obtained from running the algorithm on the JETSON NANO hardware platform. From the detected objects, the sTetro platform will plan the path to clean the staircase efficiently.

### 4.2. Experiment in Real Environment with sTetro

**Figure 10:**
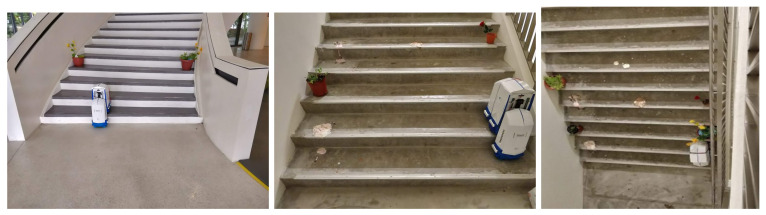
Test bed.

To evaluate the proposed scheme, the trained model was tested on three steps. These include staircase detection and localization, first step detection and obstacle and debris detection and localization. The experiment was performed in a staircase present in multistory buildings with the sTetro robot platform as shown in Figure 10.

#### Staircase Detection

**Figure 11:**
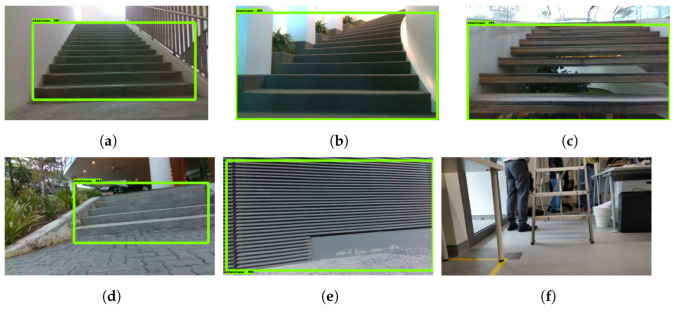
SSD MobileNet Staircase Detection Results. (**a**–**d**) Correct detections of staircases. (**e**) False detection as staircase. (**f**) Ladders not detected.

Figure 11 shows the detection result of the SSD MobileNet model for both straight and curved staircases. For the experiment’s purpose, sTetro was placed at different distances and angles from the staircase. The detection result shows that the trained model was able to detect both curved and straight staircases accurately from different distances and angles. These results are highlighted in Figure 11a–d.

**Figure 12:**
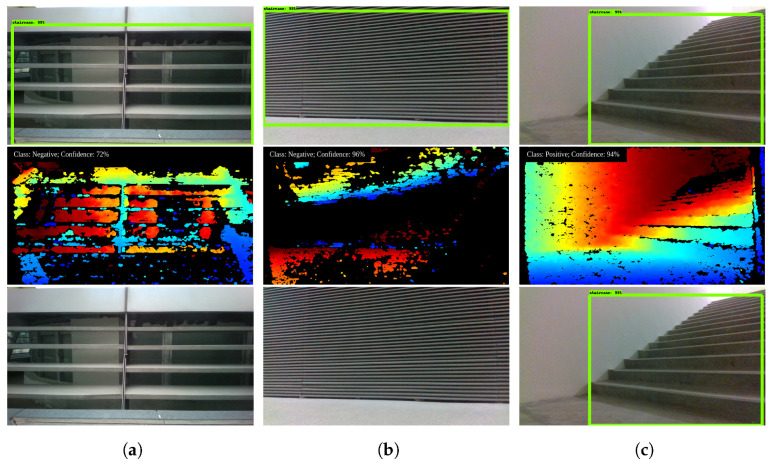
Depth based falsedetection correction. The top represents detection from SSD. Middle is the corresponding depth map. Bottom shows the results predicted by the SVM. (**a**) SVM Result: False Detection; (**b**) SVM Result: False Detection; (**c**) SVM Result: Correct Detection.

However, only the use of SSD MobileNet model has some false detections (Figure 11e). The falsely detected objects are those with RGB features similar to staircases. These include structures with parallel line textures, railings and textured walls. The results after fusion with the depth information are shown in Figure 12. Through the fusion of depth information as per the proposed scheme which uses MobileNet and SVM classifiers, these false detections are rectified. Such examples are shown in Figure 12a,b. Furthermore, the scheme also works for normal staircases (Figure 12c). This shows that the proposed detection model is able to determine staircases accurately and filter out false detections.

### 4.3. First Step Detection and Localization

**Figure 13:**
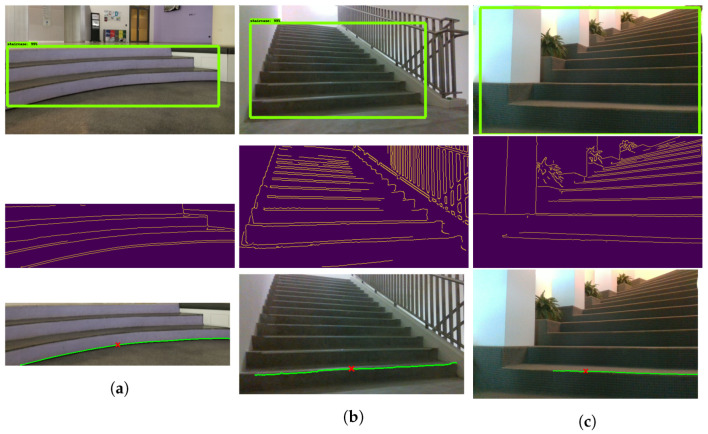
First Step Detection. From top to bottom: Staircase detection, Edge detection inside the bounding box, Contour and midpoint detection. The red cross represents the center point of the staircase. (**a**) curved stair case, (**b**) straight staircase, (**c**) cross view of straight staircase.

The next stage of the experiment was the first step detection, which was performed on the detected staircase region. The three steps involved the detection of the staircase, then detection of edges, which was followed by detection of the first step and its center. These steps for determining the first step location are shown in Figure 13. For each case, the corresponding angle of approach and distance to the first step are also described. The algorithm was able to determine the first step accurately in straight staircases (Figure 13b). Moreover, the algorithm was able to detect the first step location in textured staircases (Figure 13c), even when there could be noise generated during canny edge detection. Furthermore, the algorithm worked in curved steps, as shown in Figure 13a.

### 4.4. Obstacle Detection and Localization

**Figure 14:**
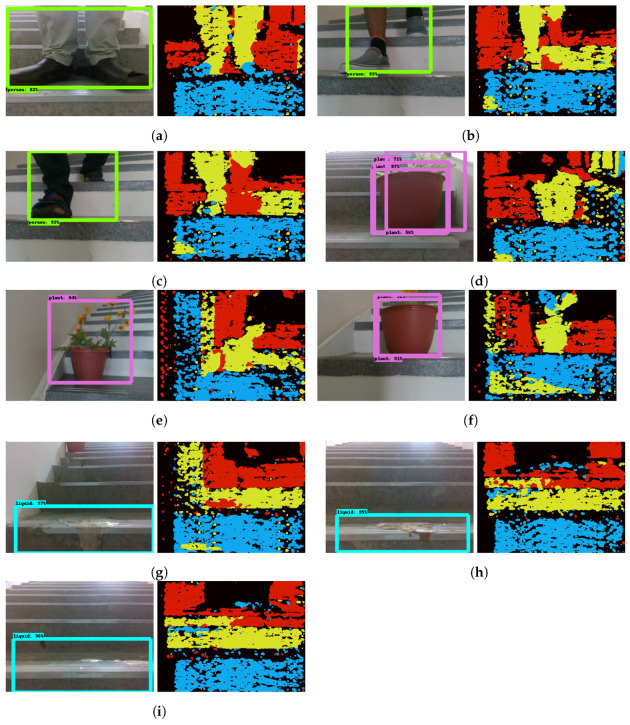
Detection and clustering results for obstacle and debris detection. Left: RGB detection, Right: Depth clustering. (**a**–**c**) Human detection; (**d**–**f**) Plant detection; (**g**–**i**) Debris detection.

The detection results of obstacles and debris on the climbing stage are shown in Figure 14. Here, obstacle detection is examined with static objects (flower/plant pots) and dynamic objects (humans walking on the steps), and debris detection with commonly found debris in staircase environments such as stains and liquid spillage. The obtained results prove that the trained model accurately detects the obstacle and debris. Furthermore, the depth based clustering and location determined for such detected obstacles is shown in Figure 14. For stain and spillage, depth data is close to the steps. However, through the clustering scheme, the framework is able to localize the debris position. This shows that the algorithm is able to detect and localize both obstacles and debris in various scenarios.

### 4.5. Results and Analysis

Table 2 and Table 3 indicate the statistical analysis of each class and detection timing (Table 4) of each stage. The proposed scheme with the fusion of depth information performs better than a standard SSD MobileNet framework for staircase detection, as illustrated in Table 2. The obtained results disclosed that there was no change in precision, but there was an improvement in recall, which was due to the decrease in false positives detected. Further, the statistical results are shown in Table 3 which indicates that the EPS detected obstacles with 92% precision and debris with 94% precision on average, which is suitable for detection applications.

Further, timing analysis (Table 4) shows that the algorithm can effectively detect the staircase with an average time of 160.30 ms, first step detection 40 ms, obstacle (includes humans and pots) and debris with an average time of 112.22 ms on the Jetson Nano hardware.

#### Comparison with Existing Scheme

The effectiveness of the proposed model is evaluated with existing staircase recognition and debris detection approach. The staircase detection approach is compared with the Unmesh et al. [26], Munoz et al. [41] and Wang et al. [14] schemes. Here, the Munoz et al. [41] and Wang [14] staircase detection schemes are employed using fusion of Hough transform and SVM classifier. In [26] the authors use the tiny-YOLOv3 CNN framework for recognizing the staircase, and Hough Transform and Statistical filtering based image processing pipeline for steps detection. Table 5 indicates the comparison results of the proposed scheme with the above-mentioned three methods. The comparative study was performed based on the detection accuracy of each model. YOLO based CNN frameworks are faster than SSD MobileNet but have a low accuracy [26]. SSD-MobileNet has shown a better trade off between accuracy and computation time. Further, removal of false detection through depth based fusion has enhanced the detection accuracy of the SSD Mobilenet framework. In contrast with non-deep learning based approaches [41] and Wang [14], our proposed algorithm outperforms the existing schemes.

In the literature, very few works have been reported for debris detection, such as yang et al.’s [42] trash classification scheme for recycling task, Rad et al.’s [43] street waste localization and classification and Gaurav Mittal’s spot garbage detection scheme [44]. However, these debris detection works were developed for different applications. Providing a fair comparison with the present scheme is very difficult. Hence, we report only the difference of the proposed debris detection scheme with an existing scheme in terms of CNN architecture and detection accuracy. Table 6 reports the difference of the proposed debris detection scheme with the existing CNN based debris detection scheme.

## 5. Conclusions

This work proposed the cascaded machine learning based operational framework for the staircase maintenance robot, sTetro. The SSD MobileNet object detection framework was utilized in the EPS to recognize the staircase, obstacles and debris. The feasibility of the proposed method was verified in real time with Jetson Nano, which is a lightweight and low power hardware. The framework detected the objects in real time and took an average of 200 ms to execute the staircase detection, first step localization and 112 ms for obstacle and debris detection. The statistical analysis shows that the depth based fusion scheme reduces the false detection rate and enhances the classification accuracy of the detection system. Further, the first step detection results ensure that the contour detection algorithm is able to determine the first step of the staircase with varying shapes and structures. Through the depth based clustering scheme, obstacles and debris position are effectively localized in the staircase. In the future, we plan to reduce the detection time of the framework by optimizing the CNN layers and also increase the number of obstacle and debris class for safe navigation and efficient cleaning on staircases.

## Figures and Tables

**Table 1:** Hardware and sensor specification.

Description	Specification	Interface
ToF sensor	SEN-02815, Range 10 cm	I2C
Vision sensor	Intel Real sense D435	USB 3.0
Bump sensor	Limit switch mechanism	Binary logic
WORM gear motor	12 volt, 100 RPM	UART

**Table 2:** Statistical analysis for staircase detection.

Model	Accuracy (%)	Precision (%)	Recall (%)
SSD MobileNet	85.23	97.72	79.24
Proposed Scheme	94.32	97.72	93.33

**Table 3:** Statistical analysis for obstacle and debris detection.

Task	Accuracy (%)	Precision (%)	Recall (%)
Obstacle Detection	93.81	92.78	94.73
Debris Detection		94.84	92.93

**Table 4:** Computation Time of Each Module.

Process	Average Execution Time in Jetson Nano (Millisecond)
Staircase Detection	110.52
SVM and MobileNet based False correction	50.21
First Step Detection	40.30
Obstacle & Debris Detection	112.22

**Table 5:** Comparison with other staircase detection schemes.

Algorithm	mAP (%)
Hough Transform and SVM [41]	92.70
Hough Transform and Two Stage SVM [14]	97.2
Yolo V2 CNN [26]	77.00
Proposed scheme	97.72

**Table 6:** Comparison with existing debris detection scheme.

Framework	Precision (%)
AlexNet [44]	87.69
11 layer CNN [42]	22.0
OverFeat GoogleNet [43]	63.2
Proposed (MobileNet SSD)	94.84

## Data Availability

Not applicable.
